# The Potential of Immune Modulation in Therapeutic HIV-1 Vaccination

**DOI:** 10.3390/vaccines8030419

**Published:** 2020-07-27

**Authors:** Nabila Seddiki, Florence Picard, Léa Dupaty, Yves Lévy, Véronique Godot

**Affiliations:** 1 Inserm, U955, Equipe 16, 94000 Créteil, France; florence.picard@inserm.fr (F.P.); lea.dupaty@gmail.com (L.D.); yves.levy@aphp.fr (Y.L.); veronique.godot@gmail.com (V.G.); 2 Faculté de médecine, Université Paris Est, 94000 Créteil, France; 3 Vaccine Research Institute (VRI), 94000 Créteil, France; 4 INSERM U955 Equipe 16, Université Paris-Est Créteil, Vaccine Research Institute (VRI), 51, Avenue du Maréchal de Lattre de Tassigny, 94010 Créteil, France; 5 AP-HP Hôpital H. Mondor—A. Chenevier, Service d’Immunologie clinique et maladies infectieuses, 94010 Créteil, France

**Keywords:** cytokines, immune checkpoint blockers, combinatorial intervention, HIV-1, therapeutic vaccine

## Abstract

We discuss here some of the key immunological elements that are at the crossroads and need to be combined to develop a potent therapeutic HIV-1 vaccine. Therapeutic vaccines have been commonly used to enhance and/or recall pre-existing HIV-1-specific cell-mediated immune responses aiming to suppress virus replication. The current success of immune checkpoint blockers in cancer therapy renders them very attractive to use in HIV-1 infected individuals with the objective to preserve the function of HIV-1-specific T cells from exhaustion and presumably target the persistent cellular reservoir. The major latest advances in our understanding of the mechanisms responsible for virus reactivation during therapy-suppressed individuals provide the scientific basis for future combinatorial therapeutic vaccine development.

## 1. Introduction

After almost forty years of HIV-1 infection, we have not been able to achieve a cure, and none of the vaccines have proven to be effective (reviewed in [1]). However, the field has established solid platforms for basic immunology, pathophysiology, and development of new immunomodulatory agents that have proven their efficacy in animal models and in vitro experiments. A new vaccine concept with the idea of mobilizing the immune system is the way to go, in order to reverse the persistent inflammation and rescue anti-HIV-1 T-cell responses that enable the clearance of the reservoir. Some immunological interventions using novel immune-cytokines, immune checkpoint blockers, and/or latency reversing agents are warranted. Similar conceptual interventions have been successfully demonstrated in the cancer field. 

Recently, some novel immuno-cytokines with improved half-life and physicochemical properties, such as IL-15 superagonists, have proven their potency. This will hopefully guide new avenues for developing combinatorial therapeutic vaccines with the objective to preserve the function of HIV-specific T cells from exhaustion and reduce the HIV-1 reservoir.

Based on the new literature, we update hereinafter some key elements for therapeutic vaccine development that could be considered in the near future and notably draw attention to their use in combination to reprogram the immune system.

## 2. Strategies for Combinatorial Therapeutic Vaccines: Potentiality of Immuno-Modulatory Agents

### 2.1. Interleukins IL-2, IL-7 and IL-15

The common γ-chain cytokines, interleukin-2 (IL-2), IL-7, IL-15, and IL-21, are critical to the physiological proliferation, regulation, function, and homeostasis of T cells [2]. IL-2, IL-7, and IL-15 are important for T cell survival and complement each other in their specific roles, from naïve T cell priming to the long-term survival of the memory state [3]. Of note, a significant fraction of circulating human memory T cells do not express IL-7R, which is likely related to their long-term survival generally mediated by IL-15 [4]. However, a selective and important expression of IL-7Ra is found on CD8^+^ memory precursor T cells, and this has led to extensive studies where IL-7 therapy has been suggested as a potent booster for vaccine responses. More importantly, we have reported that CD4^+^ Tregs express a reduced expression of IL-7Ra chain [5] but an increased expression of IL-2Ra chain [6], and this is very interesting because these two important molecules can be simultaneously targeted to potentiate effector T cells in therapeutic HIV-1 vaccination. Of note, Tregs that suppress anti-HIV-1-specific T cells need to be controlled while designing an immunotherapy in HIV-1 infection [7,8,9].

Phase I/II trials showed that administration of exogenous IL-7 was well tolerated, and significant expansions of both naïve and memory CD4^+^ T cells were observed [8,9]. IL-7 therapy only led to a small transient increase in HIV-1 DNA levels per milliliter of blood in the treated patients [10]. However, a recent study reported that rhIL-7 therapy has caused an expansion of the HIV-1 reservoir in patients defined as “poor immunological responders”, which partially decreased to baseline three months after treatment [11]. The benefit of IL-7 for CD4+ T cell reconstitution in HIV-infected patients might be reconsidered by taking into account the schedule of IL-7 administration and the need for repeated cycles in order to maintain high CD4+ T cell counts [12].

IL-15 is a highly potent and promising candidate for immunotherapy [13]. It is crucial for natural killer (NK), (Natural Killer T cells) NKT, and memory CD8+ T cell function and homeostasis. The combination of IL-15 with IL-15Ra has high biological potency with antiviral functions (reviewed in [14]). Novel IL-15-based therapies, such as N-803 (formerly ALT-803) and hetIL-15 (IL-15/IL-15 receptor α heterodimer) molecules [15], are now used in pre-clinical models, thereby overcoming limitations of free IL-15. Both molecules are particularly interesting in this regard, as their use enhanced CD8+ T cell and NK cell functions in several pre-clinical and clinical studies [15,16,17,18,19,20,21]. Interestingly, in a very recent study, it was demonstrated that subcutaneous dosing of N-803 in rhesus macaques led to robust proliferation of NK and memory CD8+ T cells with efficient cell redistribution from blood to B cell follicles in the lymph nodes, where latent virus can be found [20].

### 2.2. Immune Checkpoint Blockers (ICB)

Immune checkpoints are essential molecules that play a critical role in the regulation of immune responses. They negatively regulate T cell activation, T cell proliferation, and effector functions [22,23,24,25,26,27,28]. The blockade of immune checkpoint molecules has in fact attracted considerable interest with the aim to enhance HIV-1-specific T cell responses and decrease the HIV-1 reservoir; notably, recent studies reported high levels of viral DNA in CD4+ T cells expressing immune checkpoint molecules [29]. Ipilimumab, nivolumab, and pembrolizumab molecules, targeting cytotoxic T-lymphocyte associated antigen 4 (CTLA-4) and programmed death-1 (PD-1), respectively, have shown potent efficacy in cancer immunotherapy [30], therefore providing a solid basis for developing similar immunotherapeutic agents for HIV-1-infected individuals. There are currently ongoing clinical trials in HIV-infected cancer patients to determine the efficacy of the immune checkpoint blockade (NCT02408861, NCT03354936). New antibodies against programmed death ligand 1 (PD-L1) were also approved: atexolizumab [31], avelumab [32], and durvalumab [33]. These major advances in immunological intervention strategies are very attractive and provide the scientific basis for developing potent combinatorial therapeutics to enhance immune responses and thus gain more efficient control of virus replication.

Administration of an anti-PD-1 antibody to simian immunodeficiency virus (SIV)-infected rhesus macaques resulted in a potent expansion of virus-specific CD8+ T cells with improved functionality and led to lower SIV RNA levels in plasma [34]. Additionally, when anti-PD-1 was administered prior to antiretroviral therapy (cART), an improvement in effector T cell function with a reduction in the frequency of Tregs was reported, which resulted in better control of viremia [35]. Another study reported beneficial effects of anti-PD-1 antibody treatment with reduced interferon signaling and improved gut permeability [36]. Interestingly, a recent study by Fenwick et al. identified a novel class of antagonistic anti-PD-1 antibodies (Abs) that act independently of the PDL-1 blockade but through the CD28–AKT–NF-κB costimulatory pathway. This new class of non-blocking anti-PD-1 Abs synergized with the classical blocker anti-PD-1, i.e., Pembrolizumab, showing an improvement of antitumor activity, thus improving our understanding of the biology and function of PD-1 [37].

Combined therapy with anti-PD-1, anti-PD-L1, or anti-CTLA-4 greatly enhanced the efficacy of ALT-803 in remission of primary and advanced tumors in different pre-clinical studies [17,18,19,38,39]. Recently, N-809, a first-in-class bifunctional agent comprising the N-803 molecule fused to two anti-PD-L1 domains, has demonstrated its efficacy by providing immune-activating signals while preventing suppression in anti-tumor studies [40,41]. Moreover, a Phase I clinical trial studying the effect of spartalizumab, a humanized IgG4 monoclonal antibody that blocks binding of PD-1 to PD-L1/2, in combination with HetIL15 is being trialed in patients with metastatic cancer (ClinicalTrials.gov identifier: NCT02452268).

More recently, Bradley et al. determined that Env antibody responses to the HIV envelope could be enhanced by ICB (especially CTLA-4 blockade) with an increase in T cell proliferation [42]. Another recent study demonstrated that the dual blockade of CTLA-4 and PD-1 in cART SIV-infected rhesus macaques using monoclonal antibodies was remarkably more effective than the PD-1 blockade alone in enhancing T cell cycling and differentiation, expanding effector memory T cells and inducing robust viral reactivation in plasma and peripheral blood mononuclear cells. Moreover, in lymph nodes, this dual blockade led to decreased SIV-DNA and SIV-RNA in B cell follicles, a major site of viral persistence during cART [43]. However, none of these interventions enhanced SIV-specific CD8+ T cell responses or viral control after cART interruption. One of the latest studies in the field of HIV cures by McBrien et al. demonstrated that the administration of the IL-15 superagonist N-803 in both SIV-infected macaques and HIV-infected humanized mice induced a highly robust and persistent reversal of latency when CD8^+^ T cells were depleted [44]. These results are very intriguing and need to be demonstrated using other latency reversing agents (LRA), and they suggest a substantial role of CD8^+^ T cells in suppressing the effect of latency reversing agents such as N-803. We should thus pay attention to these new avenues while designing novel combined therapeutic vaccines.

Antibodies against other checkpoint molecules, such as LAG3, TIM3, and TIGIT, are all in early clinical development, and their use as therapeutic agents will be known in the near future [45].

## 3. Paving the Way toward Therapeutic Vaccination: Pay Attention to the Persistent Inflammation

During the course of chronic HIV-1 infection, disease progression is closely associated with a high level of immune activation, which is most likely a cause of the damage to the immune system rather than being its consequence [46]. Persistent inflammation is another important threat to disease progression, despite suppressive cART [47]. Many of the pro-inflammatory pathways, such as microbial translocation through the gut barrier [48] and the presence of common herpes viruses, are significant contributors [49,50,51,52]. This persistent inflammation can cause fibrosis in lymphoid tissues, thus leading to CD4+ T cell regenerative failure [53,54,55]. Another factor leading to persistent inflammation is type 1 interferon (IFN-I). Early IFN-I signaling during acute infection is critical to suppress virus replication, but its persistence may be detrimental and accelerate disease progression [56,57]. Several studies reported strong correlations between activated IFN-I pathways with HIV-1 or SIV disease progression [58,59,60,61,62]. Importantly, HIV-infected patients that do not develop severe disease symptoms despite high plasma viral load exhibit paradoxically low levels of IFN-stimulated-gene (ISGs) expression [63]. Moreover, despite reduced viremia in patients under cART, low levels of IFN-I persist, ISGs remain upregulated in peripheral blood cells or lymphoid organs [64,65], and administration of recombinant IFN-I showed little or no beneficial effects in HIV-1 patients [66,67,68]. These studies clearly suggest that IFN-I may contribute to increased clinical complications in HIV-1-infected and treated patients [69]. Therefore, it seems important to consider resolving the residual inflammation before attempting to redirect the immune system toward inducing a robust adaptive immunity while proposing a therapeutic vaccine, for example [70]. Therefore, treatment of infected persons using anti-inflammatory drugs may provide some benefits [71,72,73]. Blocking IFN-I signaling might be an interesting therapeutic strategy to reverse inflammation-associated disease. In this context, a nice study by Cheng et al. [74] reported that using a monoclonal antibody to bind and block IFN-α/β receptor 1 (IFNAR1) in humanized mice persistently infected with HIV-1 under effective cART, the IFNAR1 blockade fully reversed HIV-1-induced inflammation and reduced the size of HIV-1 reservoirs in lymphoid tissues. Novel therapies are warranted, and these can be tested in relevant and robust new humanized mice models.

## 4. Humanized-Mice (Hu-Mice) for Combinatorial Therapy Testing

Humanized-mice (hu-mice) used in the field of immunology present an immunodeficient background, in which an immune system of human origin is restored through the transfer of either peripheral blood cells or hematopoietic stem cells. These mice represent a unique opportunity to study in vivo pathogens infecting only human cells such as HIV and are complementary with research performed in non-human primates [75]. Currently, hu-mouse models used for HIV investigations comprise mainly three different backgrounds, the NOD-SCID IL2Rnull, NOD-Rag2-/-IL2Rnull, and Balb Rag2-/-IL2Rnull (abbreviated NSG, NRG, and BRG, respectively). The further combination of transplantation of human fetal pluripotent hematopoietic stem cells with surgical engraftment of human fetal thymic tissue and the constitutional genetic knock-out and knock-in for human cytokines or growth factors largely contributed to improve the engraftment and functionality of the human immune system in these hu-mice. These animal models have thus became valuable in vivo models for the development of vaccine strategies and therapeutic interventions [76].

The human immune cells established in hu-mice following the transfer of human hematopoietic cells can be effectively infected with HIV-1 by the same routes (mucosal or IV) as humans, and the evolution of viremia can be followed over time, correlating with the progressive loss of human CD4+ T cells in lymphoid tissues and blood. cART given to humans works effectively on these HIV-infected hu-mice, allowing a progressive human CD4+ T cell increase. Interestingly, and comparably to what has been described in humans, cART does not prevent the establishment of HIV-1 reservoir latency in hu-mice soon after infection. HIV-1 infection also causes in hu-mice a depletion of CD4+ T cells in the gut [77]. Therefore, the hu-mice models recapitulate the major features of HIV-1 infection and appear to be good models to test functional cures of HIV-1, including strategies to turn off the deleterious inflammation, to revert T cell exhaustion, and therapeutic vaccination strategies.

As mentioned above, the residual inflammation that persists in cART patients is a real threat, as it participates in the maintenance of HIV-1 reservoir. Recent studies performed on hu-mice provided new insights regarding the chronic inflammation during HIV-1 infection. In particular, one study demonstrated that chronic HIV-1 infection depletes ILC3s (innate lymphoid cells) in the gut of hu-mice through Plasmacytoid dendritic cell (pDC) activation and IFN-I production or the TLR signalling pathway [78,79] and has suggested that modulating plasmacytoid dendritic cells and IFN-I (pDC/IFN-I) to rescue ILC3s will likely be of value in preventing gut integrity. The same group also demonstrated that antibodies blocking the IFN-α/β receptor (IFNAR1) fully reversed HIV-1–induced immune hyperactivation and rescued anti-HIV-1 immune responses in T cells from HIV-1-infected hu-mice under cART [74]. Antibodies blocking IFN-α/β receptor signaling in hu-mice give similar results as compared to anti-PD-1; however, their efficacy has never been compared head to head.

Previous studies on hu-mice and in line with NHP studies showed that blocking the PD-1/PD-L1 axis with a blocking anti-PD-1 can reduce viral load by half [80], while anti-PDL1 improves CD4+ T cell levels [81]. Interestingly, a very recent study has evaluated in hu-mice a lentiviral vector-based Dendritic cell (DC) vaccine, in which HIV-1 antigen was co-expressed with CD40 ligand (CD40L) and a soluble high-affinity PD-1 dimer [82]. The idea was to improve the ex vivo DC-based vaccines already tested in HIV-infected patients with rather little success [83,84,85] by providing long-term endogenous antigen presentation, enhancing the maturation of DC through the activation of CD40, and inhibiting the PD-1/PD-L1 axis. In this proof-of-concept study, the vaccination of hu-mice with genetically modified DC induced an anti-HIV-1 immune response that suppressed viral loads as early as 2 weeks post-HIV-1 challenge and increased the function of the responding T cells [82].

The control of viral load and the improvement of T cell functions, as well as the generation of Env-specific IgG^+^ B cells, were also achieved in our hands after the vaccination of the hu-mice with DC-targeting vaccines submitted by Godot et al. [74]. Our DC-based vaccines consisted in bringing the antigens to a DC-endocytic receptor by generating a humanized anti-CD40 monoclonal antibody with the Fc domains fused to HIV antigens gag, pol, or nef peptides or the Env protein [74].

Recent studies in the field of cancer research revealed new insights into differences in response to anti-PD-1 treatment. They clearly demonstrated that the composition of the gut microbiota impacts the efficacy of anti-PD-1 or the combinatorial checkpoint inhibitor blockade [86,87]. As some HIV-1 patients suffer from dysbiosis, it is important to take into account the possible partial efficacy of such treatments in these individuals. It is therefore timely to take advantage of the availability of optimized hu-mouse models to identify biological predictors of ICB treatment efficacy but also to study the mechanisms underlying PD-1 impact on immune functions in light of the microbiota composition. However, we keep in mind and carefully consider the low reconstitution in gut-associated lymphoid tissues when interpreting experimental results for these specific questions.

With the aim of enhancing immune responses in HIV-1 infection induced by vaccination, hu-mice have also proved to be relevant models to evaluate other immunomodulatory approaches like the use of TLR agonists or recombinant interleukins. Hu-mice have been useful in demonstrating that a combination of a TLR3 agonist and a CD40-targeting vaccination could enhance HIV-1-specific cellular responses but also reduce viral reservoirs [74]. As we saw above, IL-2, IL-7, and IL-15 are promising candidates to modulate the function of T cells during HIV-1 infections. However, these cytokines are also crucial in the commitment and development of NK cells. The NK cells are key players of the innate immune responses during viral infection through their cytotoxic functions, whose potency in HIV-1 virulence control has also been reported [88]. In hu-mice, the administration of recombinant huIL-15, or vector expressing huIL-15 or IL-15 coupled to IL-15Rα (the IL-15/IL-15Rα superagonist as described above), increased the number of NK cells [89,90]. The IL-15 superagonist injected concomitantly with HIV-1 inoculation almost completely prevented infection in hu-mice [38]. Even though the IL-15 superagonist does not seem to affect the viral latency in NHP models [20], it can boost the effect of latency reversing agents in vitro [91]. Therefore, its potential remains interesting, as it can boost the immune responses at an early time point or during cART.

We also mentioned above that the use of recombinant human IL-7 (rhIL-7) could be another interesting way to boost and modulate immune responses prior to vaccination [8,10,92]. Studies performed in hu-mice showed that comparable to humans, the administration of a lentiviral vector coding for hIL-7 or the injection of rhIL-7 expanded adoptively transferred hematopoietic stem cells [93,94]. However, consistently with what was found in human studies, rhIL-7 did not affect viral loads in hu-mice [95].

As mentioned above, HIV-1 reservoirs are a major barrier in overcoming HIV-1 infection, and LRA represent a class of drugs used in the “shock and kill” therapeutic strategy to overcome the viral latency [96]. LRA comprise various types of molecules, including some blocking antibodies such as anti-IFN-I, anti-PD-1, or TLR3 agonist [97]. Interestingly, recent studies performed in humanized myeloid only mice (MoM) have revealed the relevance of hu-mouse models in this area of HIV research. Indeed, these hu-mouse studies demonstrated that myeloid cells, comprising the microglial cells, may represent a significant reservoir for HIV-1 latency [98,99]. Since then, another study confirmed that the development of the hu-mouse models offers an opportunity to investigate the establishment of the myeloid HIV reservoir and the effect of LRA on its clearance [100].

**Figure 1:**
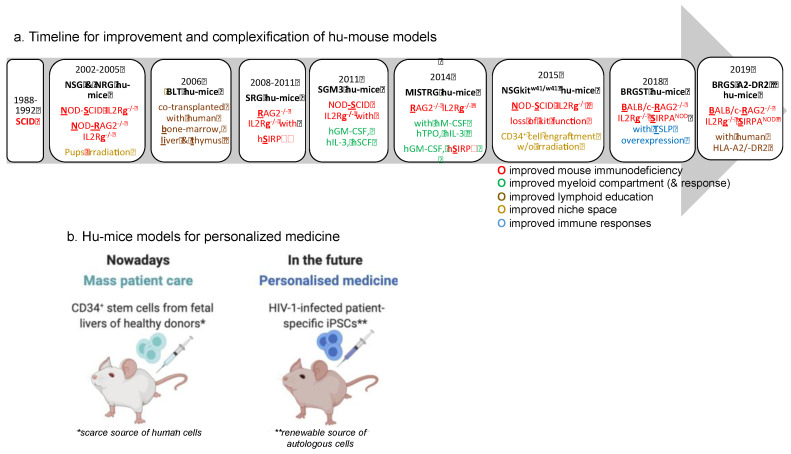
Hu-mice models for personalized medicine: myth or reality. (**a**) Timeline of major improvements in the generation of hu-mice reconstituted with a human immune system. The name of the different hu-mouse models is given in black and bold. Their genetic background is indicated as well as their other genetic modifications or alterations. (**b**) Hu-mice models were improved for Human HLA-I and human HLA-II, myeloid cell engraftment including DC, lack of Graft versus host Disease (GvHD), or human B cell functions, including higher levels of antigen-specific human antibodies.

The potential for further advances in the discovery of new HIV treatment options provided by numerous studies using humanized mouse models remains promising. The generation of new humanized mice based on the use of immunodeficient Il2rg–/– hosts combined with additional genetic modifications (summarized in Figure 1a), including the expression of human cytokines and/or growth factors, has overcome many of the limitations of previously available models. However, the actual hu-mouse models retain some limitations; in particular, we are still missing a hu-mouse model with both cellular and humoral improved responses. In the future, we could imagine the use of HIV patient-specific pluripotent stem cells (iPSCs) to reconstitute the immune system of hu-mice instead of CD34^+^ stem cells from healthy fetus and thus move toward a more specific and personalized medicine (Figure 1b).

## 5. Conclusions

**Figure 2:**
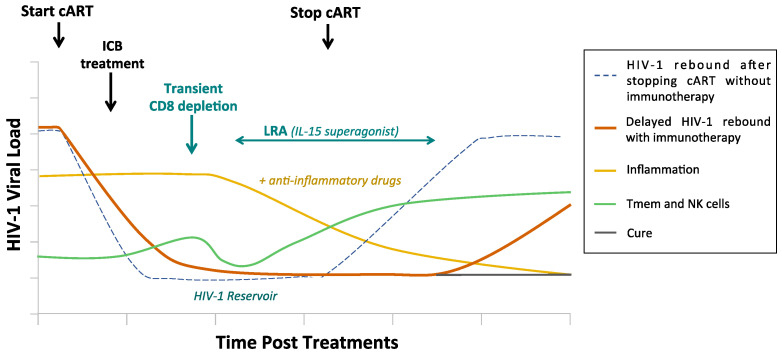
Combinatorial therapeutic approach in HIV-1 infection. The persistent inflammation and virus reservoir during HIV-1 infection in the presence of antiretroviral therapy (cART) contribute to HIV-1 disease progression. The use of combinatorial therapy such as: (1) Immune checkpoint blockers (ICB) to reverse cell exhaustion and boost memory cell responses; (2) anti-inflammatory drugs to reduce the persistent inflammation and unmask reinvigorated T cell responses; (3) latency reversing agents (LRA) (IL-15 superagonists); and CD8+ T cell depletion to reverse latency and render the virus visible to the immune system.

Almost forty years of HIV research have provided many insights, but there are still remaining questions that pose challenges to curing HIV. Combinatorial interventions for developing novel HIV-1 therapeutic vaccines are on the way. Treatment using ICB and/or recombinant cytokines have shown promising results in animal models and our understanding of the mechanisms responsible for virus reactivation during cART-suppressed infection is growing. However, further studies are warranted, as we need to translate these findings into humans. Additionally, it will be important to consider resolving the persistent residual inflammation that has largely been documented in cART-suppressed individuals and is considered a serious ingredient that maintains lately infected cells. This will be crucial if one wants to redirect the immune system towards inducing a robust adaptive immunity when giving a therapeutic vaccine. Clinical trials using ICB, anti-inflammatory drugs, and some promising molecules, such as IL-15 superagonists in combination with a potential CD8^+^ T cell depletion (Figure 2), should build the next important outcomes in the field for achieving a true sterilizing cure for HIV.
